# Bone structure and body composition in adolescents with cow’s milk allergy in infancy: a clinical cohort study

**DOI:** 10.1136/bmjpo-2025-004087

**Published:** 2026-01-21

**Authors:** Sonja Piippo, Tero Varimo, Helena Hauta-alus, Mirva Viljanen, Erkki Savilahti, Outi Mäkitie, Mikael Kuitunen

**Affiliations:** 1 Children's Hospital, Pediatric Research Center, University of Helsinki and Helsinki University Hospital, Helsinki, Finland; 2 Research Program for Clinical and Molecular Metabolism (CAMM), Faculty of Medicine, University of Helsinki, Helsinki, Finland; 3 Clinical Medicine Research unit, MRC Oulu, Oulu University Hospital and University of Oulu, Oulu, Finland

**Keywords:** Adolescent Health, Endocrinology

## Abstract

**Objective:**

We compared bone structure and body composition in adolescents with a challenge-confirmed cow’s milk allergy (CMA) during infancy to peers with refuted CMA and to controls.

**Design:**

An observational clinical cohort study.

**Setting:**

A tertiary allergy clinic at Helsinki University Hospital.

**Patients:**

From a randomised controlled trial from 1999 to 2001 evaluating the effect of probiotics on atopic eczema, we followed up participants (n=81) at 15–18 years’ age and recruited age-matched controls (n=49). Original study participants all had atopic eczema, and CMA confirmed (n=43) or refuted (n=38) by double-blind placebo-controlled cow’s milk challenge in infancy.

**Main outcome measures:**

The primary outcome was differences in volumetric bone mineral density (vBMD) measured with peripheral quantitative CT. Secondary outcomes were differences in body composition by a bioelectric impedance analysis.

**Results:**

Participant’s median age was 17.3 years, 62% were females. After adjusting for sex, age-adjusted body mass index, past 5 years’ mean daily supervised physical activity, daily vitamin D intake from food and supplements and mean daily intake of dairy products, the CMA-confirmed group had, compared with the CMA-refuted group, lower median total vBMD at the distal radius (Z-scores −1.49 vs −0.78). The CMA-confirmed group had lower median total vBMD (Z-scores −0.05 vs +0.01) and lower median trabecular vBMD (Z-scores +0.20 vs +0.51) at the distal tibia compared with controls. No group differences in body composition were found.

**Conclusion:**

An early childhood history of CMA may be associated with lower adolescent radial and tibial vBMD. Further studies are needed to assess this potential association.

**WHAT IS ALREADY KNOWN ON THIS TOPIC**

- In children and female adolescents with persistent cow’s milk allergy (CMA), both dual X-ray absorptiometry and high-resolution peripheral quantitative CT have found reduced bone mineral density.

**WHAT THIS STUDY ADDS**

- In milk-tolerant adolescents, a history of CMA during infancy was associated with reduced volumetric bone mineral in the radius and the tibia, as measured by peripheral quantitative CT, compared with controls.

**HOW THIS STUDY MIGHT AFFECT RESEARCH, PRACTICE OR POLICY**

- Starting a milk-elimination diet in early childhood is warranted only when CMA is challenge confirmed with clinically relevant symptoms. Reassessment for milk tolerance is fundamentally important, and when tolerance has been achieved, milk should be actively introduced.

## Introduction

 In Europe, food challenge-verified cow’s milk allergy (CMA) affects 0.3%–4.9% of children.^1 2^ Spontaneous resolution of CMA is observed in up to 79%–95% by age 15–16 years.^3 4^ CMA is treated with a milk elimination diet, which most (85%) Finnish children follow with an extreme accuracy.^5^ Studies show, in children with CMA, a compromised intake of calcium,^6 7^ which is the strength-providing mineral of bones.

Cow’s milk contains more nutrients (eg, calcium, vitamin D and protein) valuable for bone health per unit of energy than many other food groups.^8^ Adequate intake of calcium is associated with greater bone mineral density (BMD) in childhood.^9 10^ Childhood and adolescence are crucial periods for bone mineral accrual, as peak bone mass is acquired at the end of the second decade of life.^11^ For the lifetime risk of osteoporosis, a condition characterised by a reduced BMD, peak bone mass is a major risk determinant.^12^

Studies on allergic children show detrimental influence of a milk elimination diet on bone health. In children with persistent CMA, a reduced dual X-ray absorptiometry (DXA)-derived areal BMD (aBMD)^13^ and an increased risk for fractures^14^ have been found. In contrast to DXA, peripheral quantitative CT (pQCT) differentiates cortical and trabecular bone and provides a volumetric BMD (vBMD), which is not affected by the child’s size. A recent study on female adolescents with persistent CMA found in the distal tibia, with high-resolution pQCT, lower cortical thickness and lower total and trabecular vBMD.^15^

Previous studies have focused on participants with persistent CMA, a rare phenomenon.^2^ We have carried out a prospective study on children with the most common form of CMA, starting in infancy and achieving tolerance before adolescence.^16^ To the best of our knowledge, studies assessing the effect of an early childhood milk elimination diet on pQCT measured bone structure in adolescence are lacking. The effects of CMA on body composition have rarely been explored.

To assess bone structure and body composition in adolescents with a history of a milk elimination diet in infancy, we conducted a pQCT scan and a bioelectrical impedance analysis. Additionally, participant fracture rate was recorded. We compared this CMA-confirmed group to adolescents with refuted CMA and age-matched controls.

## Materials and methods

### Study design and participants

**Figure 1:**
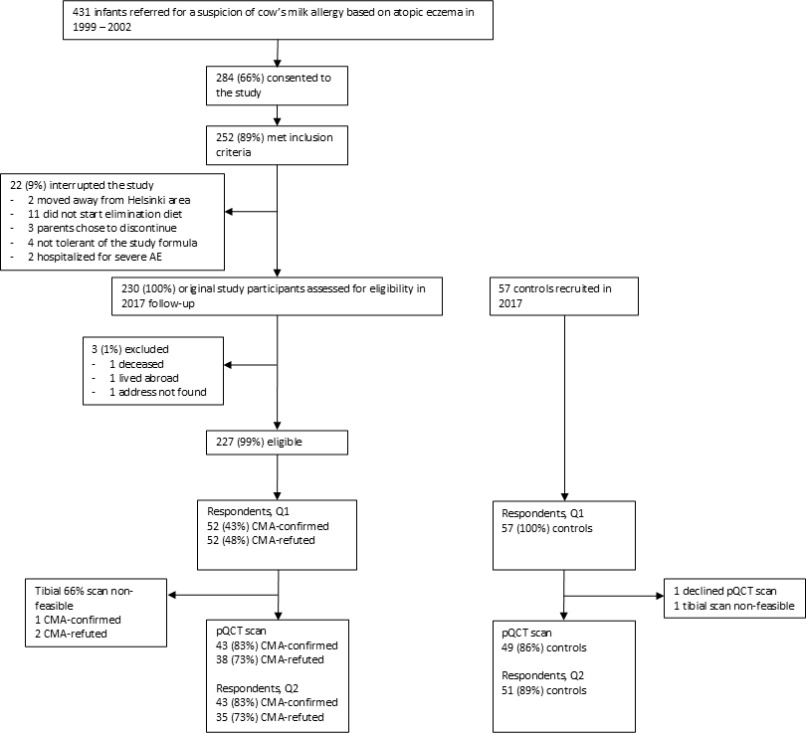
Flowchart of the study. Inclusion criteria for the original study were: 1. age<12 months at the beginning of the study; 2. symptoms indicating CMA, AE being the required symptom; 3. no routine use of probiotic preparations (daily for longer than 1 week within 6 weeks prior to the beginning of the study). AE, atopic eczema; CMA-confirmed, cow’s milk allergy confirmed—positive, double-blind, placebo-controlled, oral cow’s milk challenge during the original study; CMA-refuted, negative, double-blind, placebo-controlled, oral cow’s milk challenge during the original study; Q1, questionnaire one; Q2, questionnaire two.

In 1999–2001, a randomised, controlled trial assessed the treatment effect of 4 weeks of probiotics on atopic eczema (AE) in infants. We followed up the participants (n=230) at age 15–18 years. As three were unreachable, we contacted 227 participants. Among these, 46% (n=104) consented to our follow-up study (figure 1). Original study participants with AE and suspected CMA had been referred to a tertiary allergy clinic at Helsinki University Hospital to confirm or rule out CMA before the age of 12 months. CMA, IgE-mediated and non-IgE-mediated, was diagnosed in 120 (52%) of study participants by a double-blind placebo-controlled oral food challenge. A detailed protocol of the original study has been published.^17^ We recruited age-matched controls (n=57) by collaboration with school healthcare nurses in Helsinki, and social media advertising. One subject (control) declined the pQCT scan. We performed scans on altogether 130 participants who attended a study visit: 43 on those with confirmed CMA during infancy, 38 on those with a negative CMA challenge (CMA-refuted) and 49 controls. Due to lower extremity size, one tibial scan was unfeasible, and for three participants only distal (6%) and diaphyseal (38%) scans were obtained.

### Patient and public involvement

Patients or the public were not involved in the design, conduct, reporting or dissemination plans of this study.

### Questionnaire data

Information on physical activity (PA), bone fractures, family history of osteoporosis and the year in which cow’s milk allergic participants reintroduced milk into their diet came from a study questionnaire. PA was recorded separately for the past 12 months and 5 years, including daily activities (eg, walking or cycling to school), and both supervised and unsupervised PA. An average exercise duration in minutes per day was calculated based on reported type, frequency and duration of exercise. For each fracture, we recorded the affected bone, trauma mechanism and year. Two food frequency questionnaires collected data on dairy consumption and intake of vitamin D from food and supplements, as well as adherence to special diets.^16^

### Register data

The National Pension Institute (Kela) registers all pharmacy purchases of doctor-prescribed medicines. It grants special reimbursements for asthma medication, which patients apply for after 6 months of regular inhaled corticosteroid (ICS) use. We acquired data on purchase of ICS and asthma special reimbursement status for years 1999–2017.

### Anthropometric variables and biochemical analyses

Participants were evaluated at a study visit by a research physician (SP) and a research nurse (RP). A wall-mounted stadiometer measured height to the nearest 1 mm. A hand-to-foot bioelectrical impedance analysis with InBody 770 (InBodyUSA, Cerritos, California) measured weight, body fat percentage, fat mass and fat-free mass. Length and weight were converted into SD scores and age-adjusted body mass index (BMI-for-age), respectively, based on Finnish paediatric growth references,^18^ as previously published.^16^ The research physician clinically assessed pubertal status by Tanner staging.^19 20^

A blood sample was drawn after overnight fasting. With the standard technique, the accredited Central Laboratory of Helsinki University Hospital analysed plasma alkaline phosphatase, inorganic phosphate and calcium. Serum 25-hydroxyvitamin D was analysed with an immunoassay (fully automated) (IDS-iSYS; Immunodiagnostic System).^16^

### pQCT bone assessment

At the study visit, the non-dominant radius and the left tibia were assessed by pQCT to determine vBMD and bone geometry (Stratec XCT 2000 L Research, Stratec Medizintechnik GmbH; software V.6.20). Bone length was measured with a ruler at 0.5 cm accuracy: the radius from the ulnar styloid process to the olecranon, and the tibia from the medial malleolus to the medial condyle. A scout view established the measurement site. With a 0.5 mm voxel size, scans were obtained at 4% and 66% sites for the radius and 4%, 38% and 66% sites for the tibia. The manufacturer’s macro-based automated data interpretation software analysed the pQCT images. At the distal (4%) sites, the analysed bone parameters comprised bone mineral content (BMC), total cross-sectional area (CSA), total vBMD and trabecular vBMD. At the diaphyseal (38%) and proximal (66%) sites, these parameters were BMC, total CSA, total vBMD, cortical CSA, cortical vBMD, cortical thickness, periosteal and endocortical circumference, and polar stress–strain index. Age-adjusted and sex-adjusted Z-scores were calculated based on previously reported equations (Z-score=(Ln (X/M))/S where X is the acquired raw value, Ln is the natural logarithm and M is the mean (radius) or median (tibia) and S is the coefficient of variation).^21^,^23^ As such equations were not available for the 66% tibial measurement site, we analysed raw values. A trained and experienced research nurse performed the pQCT scans.

### Statistical analyses

Differences in categorical background characteristics between the CMA-confirmed group, the CMA-refuted group and controls were analysed by χ^2^, except for family history of osteoporosis, which was analysed by Fisher’s exact test. Normality of variable distributions was estimated by visual inspection of histograms and Kolmogorov-Smirnov test. Bone parameter Z-scores were non-normally distributed, and non-transformable and thus analysed by Kruskal-Wallis. Bone parameters from pQCT are presented as medians and IQR. Missing data were handled using pairwise deletion, allowing all available data to be used in each analysis.

Analysis of covariance (ANCOVA) was performed to assess the association between a history of CMA during infancy on vBMD in adolescence while controlling for covariates. The final model included the covariates sex, BMI-for-age, supervised PA during the past 5 years, daily dairy product consumption and daily intake of vitamin D from food and supplements. Pairwise comparisons were Bonferroni-corrected. No interaction was observed between study group and sex. Homogeneity of regression slopes, equality of error variances, multicollinearity and normality of residual distribution were analysed. These assumptions were mostly met, and the test was considered robust for minor violations.

To assess the congruence of the adjusted model for pQCT Z-scores, we also examined raw values of all parameters by ANCOVA. The differences between groups remained roughly similar (online supplemental tables 1 and 2). Spearman’s rank correlation explored associations between height and vBMD.

Statistical analyses were conducted with SPSS for Windows, V.25 and 29 (IBM, Armonk, New York). Significance was set at p<0.05. We applied a Bonferroni correction for multiple comparisons.

## Results

### Participant characteristics

Baseline characteristics are shown in table 1. The CMA-confirmed group included 43, the CMA-refuted group 38 and the control group 49 participants. The CMA-confirmed group included 24 participants (56%) with IgE-mediated CMA and 19 participants (44%) with non-IgE-mediated CMA. The median age in all study groups was 17.3 years. A higher proportion of the control group was female (82%) compared with the CMA-confirmed group (47%) and the CMA-refuted group (53%). As previously reported,^24^ from the original study cohort, male participants (p=0.004) and participants who during the original study had a mother who smoked (p=0.024) were more likely to be lost to follow-up. Based on the food frequency questionnaires, one participant in the CMA-confirmed group adhered to a milk elimination diet at the time of the present study. There was no difference in total dairy product consumption between the groups (table 1).^16^ A subanalysis within the CMA-confirmed group revealed that participants with a history of IgE-mediated CMA had a lower median total daily dairy product consumption compared with those with a history of non-IgE-mediated CMA (317 g/day vs 661 g/day, respectively) (p=0.04). In the CMA-confirmed group, 60% (n=26) of participants reported the year in which milk was reintroduced in their diet. The median duration of the elimination diet was 2 years in this group, the range being 0–11 years. Family history of osteoporosis in a second degree relative, and fracture history were similar across groups. In girls and boys, 80 (96%) and 45 (98%), respectively, had reached Tanner stage 5 of pubertal development. There was no significant difference in Tanner stage between the study groups. The CMA-refuted group had a significantly higher daily total PA over the past 5 years, compared with controls (p=0.024), and alkaline phosphatase was significantly higher in the CMA-confirmed group, compared with the control group (p=0.005). These differences were not significant after adjustment for sex. There was no significant difference in the frequency of special reimbursements for asthma medication. The CMA-confirmed group had, however, significantly more often purchased ICS during their lifetime (54%) compared with the CMA-refuted group (21%) and controls (20%).

**Table 1:** Participant characteristics

	CMA-confirmed (n=43)	CMA-refuted (n=38)	Controls (n=49)	P	P*
Age, years, median (q1, q3)	17.3 (16.9, 17.5)	17.3 (16.9, 17.5)	17.3 (16.5, 17.9)	0.95	0.83
Sex, female, median (q1, q3)	20 (47)	20 (53)	40 (82)	**0.001**	
Height, cm, mean (SD)	173.4 (10.3)	172.0 (9.4)	170.6 (8.2)	0.33	0.38
Height, SD score, mean (SD)	0.04 (1.2)	−0.05 (1.3)	0.33 (1.2)	0.30	0.41
Weight, kg, median (q1, q3)	65.7 (57.7, 73.8)	64.0 (58.1, 78.0)	62.8 (53.1, 69.0)	0.18	0.87
Age-adjusted body mass index (kg/m^2^), median (q1, q3)	22.2 (20.1, 24.3)	22.0 (19.9, 25.2)	21.0 (19.8, 22.9)	0.26	0.48
Tanner stage of puberty†				0.10	
M or G 4, n (%)	0 (0)	3 (8)	1 (2)		
M or G 5, n (%)	42 (100)	34 (92)	48 (98)		
Special diet‡, any, yes, n (%)	11 (29)	7 (23)	20 (44)	0.13	
Body fat (%), median (q1, q3)	17.9 (10.8, 28.1)	18.8 (12.7, 26.0)	20.7 (16.2, 25.6)	0.34	
Body fat mass (kg), median (q1, q3)	10.3 (6.5, 17.3)	11.2 (7.6, 18.8)	12.3 (9.2, 15.3)	0.66	
Fat free mass (kg), mean (SD)	54.3 (11.8)	52.5 (11.1)	48.8 (8.9)	**0.04**	0.874
Osteoporosis in a second-degree relative, yes, n (%)	5 (12)	4 (11)	5 (10)	1.00	
Fractures§, ever—yes, n (%)	14 (27)	13 (25)	18 (32)	0.74	
Reimbursement for asthma medication, yes, n (%)	10 (23)	4 (11)	6 (12)	0.23	
Prescription of inhaled corticosteroids, ever, yes, n (%)	23 (54)	8 (21)	10 (20)	**0.001**	
Total dairy consumption¶ (g/d), median (q1, q3)	452 (172, 1229)	566 (277, 1023)	230 (78, 800)	0.07	0.95
Serum 25-hydroxyvitamin D concentration (nmol/l), median (q1, q3)	76.0 (58.9, 97.9)	79.3 (65.1, 89.6)	81.1 (65.9, 93.6)	0.83	0.35
Plasma alkaline phosphatase**, median (q1, q3)	95 (73, 139)	94 (66, 119)	76 (61, 96)	**0.005**	0.12
Physical activity†† (min/days), median (q1, q3)					
Past 12 months, supervised	19 (0, 61)	16 (1, 44)	11 (0, 42)	0.73	0.84
Past 12 months, total	62 (28, 138)	66 (40, 103)	57 (41, 83)	0.60	0.83
Past 5 years, supervised	29 (13, 63)	28 (8, 49)	28 (12, 61)	0.33	0.42
Past 5 years, total	67 (40, 118)	80 (47, 125)	65 (46, 84)	**0.02**	0.07

P values were calculated with ANOVA or the Kruskal-Wallis test, as appropriate.

Categorical P values were calculated with the χ2 test.

Age-adjusted BMI, body fat mass and physical activity during past 12 months and 5 years; both supervised and total were transformed with natural log to achieve a normal distribution.

Statistical significance was set at P < 0.05. Significant P-values are bolded.

* Analaysis of covariance adjusted for sex.

† Missing cases: CMA-confirmed n=1, CMA-refuted n=1.

‡ Missing cases: CMA-confirmed n=5, CMA-refuted n=7, control n=3.

§ n= 159.

¶ Missing cases: CMA-confirmed n=8, CMA-refuted n=6, control n=3.

** Missing cases: CMA-refuted n=1, control n=2.

†† Missing cases: CMA-confirmed n=5, CMA-refuted n=6, control n=3.

BMI, body mass index; CMA, cow’s milk allergy.

### Comparison of pQCT parameters

**Figure 2:**
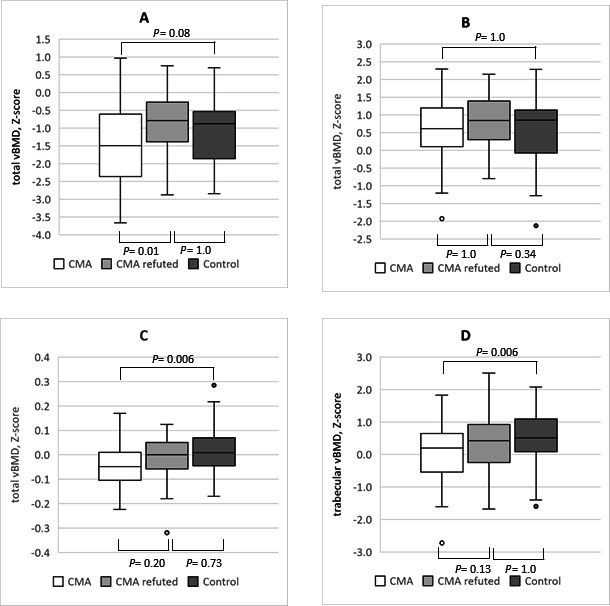
Volumetric bone mineral density (vBMD) Z-scores in the three study groups; CMA-confirmed (n=43), CMA-refuted (n=38) and control (n=49, for tibial measurements n=48): (**A**) the distal radius; (**B**) the proximal radius (**C**) total vBMD at the distal tibia and (**D**) trabecular vBMD at the distal tibia. Bonferroni-corrected significant group differences from the analysis of covariance which adjusted for sex, BMI-for-age, supervised physical activity during the past 5 years, daily dairy product consumption and daily intake of vitamin D combined from food and supplements are denoted with the appropriate P value. BMI, body mass index; CMA, cow’s milk allergy.

Bone structure findings from the radius are presented in table 2, and from the tibia in table 3. Total vBMD was the lowest in the CMA-confirmed group at all measurement sites in both the radius and the tibia, though this difference was not significant at the proximal radius and tibia. In the adjusted model in the CMA-confirmed group, total vBMD was significantly lower at the distal radius compared with the CMA-refuted group, and in the distal tibia compared with the control group (figure 2). In the distal radius, but not at other measurement sites, the proportion of vBMD Z-scores below −2.0 was higher in the CMA-confirmed group (37.2%) compared with the CMA-refuted (5.3%) and control groups (14.6%) (p=0.001). Trabecular vBMD in the distal tibia was lower in the CMA-confirmed group compared with the control group (figure 2). Cortical vBMD in the diaphyseal tibia was lower in the CMA-confirmed group compared with the CMA-refuted group (table 3).

**Table 2:** Bone structure in the radius among adolescents with atopic eczema and confirmed cow’s milk allergy during infancy, adolescents with atopic eczema and refuted cow’s milk allergy during infancy, and controls

	CMA-confirmed (n=43)	CMA-refuted (n=38)	Controls (n=49)	P	P*
Distal radius (4% site)					
Total BMC	0.12 (-0.39, 0.93)	0.37 (0.05, 0.95)	0.27 (-0.54, 1.49)	0.42	**0.005** †
Total CSA	1.38 (0.76, 2.10)	1.27 (0.71, 1.70)	1.64 (0.72, 2.16)	0.17	0.09‡
Total vBMD	−1.49 (−2.36, –0.61)	−0.78 (−1.39, –0.27)	−0.88 (−1.86, –0.53)	**0.01**	**0.009** §
Trabecular vBMD	−0.37 (−1.18, 0.75)	0.53 (−0.52, 1.01)	0.07 (−0.77, 0.99)	0.13	0.06
Proximal radius (66% site)					
Total BMC	0.41 (−0.41, 1.04)	0.23 (−0.41, 0.69)	0.20 (−0.49, 0.93)	0.90	0.08
Total CSA	0.70 (−0.23, 1.37)	0.39 (−0.40, 0.93)	0.49 (−0.16, 1.29)	0.49	**0.02** ¶
Total vBMD	0.61 (0.11, 1.19)	0.85 (0.30, 1.39)	0.85 (−0.07, 1.14)	0.32	0.09
Cortical CSA	0.31 (−0.59, 0.91)	0.12 (−0.52, 0.62)	0.21 (−0.52, 0.96)	0.83	0.31
Cortical vBMD	0.95 (0.67, 1.29)	1.23 (0.77, 1.62)	0.83 (0.18, 1.61)	0.18	0.12
Polar SSI	0.53 (−0.81, 1.14)	0.04 (−0.53, 0.64)	0.22 (−0.62, 0.94)	0.63	**0.03** ¶

Presented as Z-score medians (q1, q3).

Statistical significance was set at P < 0.05. Significant P-values are bolded.

* Analysis of covariance adjusted for sex, supervised exercise during the past 5 year, age-adjusted body mass index, mean daily intake of dairy products and mean daily intake of vitamin D from food and supplements. Missing cases: CMA-confirmed n=8, CMA-refuted n=6, control n=3.

† Pairwise comparison CMA-confirmed vs control, Bonferroni corrected p<0.05.

‡ Violates assumption of homogeneity of regression slopes.

§ Pairwise comparison CMA-confirmed vs CMA-refuted, Bonferroni corrected p<0.05.

¶ Pairwise comparison CMA-refuted vs control, Bonferroni corrected p<0.05.

BMC, bone mineral content; CMA, cow’s milk allergy; CSA, cross-sectional area; SSI, stress-strain index; vBMD, volumetric bone mineral density.

**Table 3:** Bone structure in the radius among adolescents with atopic eczema and confirmed cow’s milk allergy during infancy, adolescents with atopic eczema and refuted cow’s milk allergy during infancy, and controls

	CMA-confirmed (n=43)	CMA-refuted (n=38)	Controls (n=48)	P	P*
Distal tibia (4% site) - Z-scores					
Total BMC	−0.06 (−0.40, 0.34)	0.08 (−0.22, 0.43)	0.28 (−0.34, 0.93)	0.11	**<0.001†‡**
Total CSA	0.56 (−0.13, 1.11)	0.27 (−0.47, 0.95)	0.31 (−0.45, 1.07)	0.34	**0.017‡**
Total vBMD	−0.05 (−0.10, 0.01)	0.00 (−0.06, 0.05)	0.01 (−0.04, 0.07)	**0.01**	**0.008†**
Trabecular vBMD	0.20 (−0.54, 0.64)	0.43 (−0.25, 0.93)	0.51 (0.08, 1.09)	0.07	**0.008†**
Diaphyseal tibia (38% site)—Z-scores					
Total BMC	−0.58 (−1.32, 1.01)	−0.18 (−0.95, 0.38)	0.22 (−0.89, 0.86)	0.16	**<0.001†‡**
Total CSA	−1.25 (−2.37, –0.30)	−1.32 (−2.26, –0.37)	−0.71 (−1.69, 0.21)	**0.04**	**0.008‡**
Cortical thickness	0.87 (−0.37, 2.29)	1.09 (0.04, 2.13)	1.33 (0.73, 1.85)	0.39	**0.03†§**
Cortical vBMD	0.51 (0.14, 0.94)	1.02 (0.48, 1.40)	0.86 (0.41, 1.26)	**0.02**	**0.04¶**
Endosteal circumference	−1.67 (−2.09, –0.91)	−1.96 (−2.48, –1.17)	−1.30 (−2.25, –0.72)	0.09	0.07
Periosteal circumference	−1.22 (−2.63, –0.29)	−1.42 (−2.43, –0.36)	−0.70 (−1.62, 0.22)	**0.03**	**0.014‡**
Proximal tibia (66% site)—raw values					
Total BMC, mg/mm	381 (367–396)	388 (372–404)	420 (403–437)	0.99	**<0.001†‡**
Total CSA, mm^2^	601 (575–627)	585 (556–613)	654 (624–685)	0.66	**0.001†‡**
Total vBMD, mg/cm^3^	644 (624–664)	671 (648–693)	647 (623–670)	0.08	0.13§
Cortical CSA, mm^2^	292 (281–304)	298 (285–310)	325 (311–338)	0.96	**0.001†‡**
Cortical vBMD, mg/cm^3^	1109 (1103–1116)	1121 (1114–1128)	1113 (1105–1121)	**0.02**	0.047**
Cortical thickness, mm	3.95 (3.81–4.09)	4.11 (3.95–4.27)	4.20 (4.03–4.37)	0.60	0.016
Endosteal circumference, mm	61.5 (59.4–63.6)	59.5 (57.1–61.8)	63.9 (61.4–66.4)	0.27	**0.009‡**
Periosteal circumference, mm	86.3 (84.4–88.2)	85.3 (83.2–87.3)	90.3 (88.1–92.5)	0.66	**<0.001†‡**
Polar SSI, mm^3^	2377 (2234–2520)	2374 (2216–2531)	2714 (2546–2883)	0.90	**0.003†‡**

Presented as Z-score medians (q1, q3) for distal and diaphyseal measurement sites. Presented as means of raw values (95% CI) for the proximal measurement site.

Statistical significance was set at P < 0.05. Significant P-values are bolded.

* Analysis of covariance adjusted for sex, supervised exercise during the past 5 year, age-adjusted body mass index, mean daily intake of dairy products, and mean daily intake of vitamin D from food and supplements. Missing cases: CMA-confirmed n=8, CMA refuted n=8, control n=3.

† Pairwise comparison CMA-confirmed vs control, Bonferroni corrected p < 0.05.

‡ Pairwise comparison CMA-refuted vs control, Bonferroni corrected p < 0.05.

§ Violates assumption of homogeneity of regression slopes.

¶ Pairwise comparison CMA-confirmed vs CMA-refuted, Bonferroni corrected p < 0.05.

** Pairwise comparisons non-significant.

BMC, bone mineral content; CMA, cow’s milk allergy; CSA, cross-sectional area; SSI, stress-strain index; vBMD, volumetric bone mineral density.

In the adjusted model, BMC was significantly lower in the CMA-confirmed group compared with controls at the distal radius, and in both the CMA-confirmed group and the CMA-refuted group compared with the control group at all three tibial measurement sites (table 3). At the diaphyseal tibial site, BMC Z-score was more frequently below −2.0 both in the CMA-confirmed group (11.6%) and the CMA-refuted group (13.2%), compared with the control group (0%) (p=0.038). A subanalysis within the CMA-confirmed group found that participants with IgE-mediated CMA had a lower median Z-score compared with those with non-IgE-mediated CMA for distal radial trabecular vBMD (−0.59 vs 0.32, p=0.03), the distal tibial total vBMD (−0.08 vs −0.01, p=0.01) and trabecular vBMD (−0.18 vs 0.46, p=0.01).

In the adjusted model, the total CSA was smaller in the CMA-refuted group compared with controls in the proximal radius as well as all three tibial measurement sites. The CMA-confirmed group exhibited a smaller CSA compared with controls in the proximal tibia (table 3).

In the adjusted model, the endocortical circumference at the proximal tibial measurement site was significantly smaller in the CMA-refuted group compared with the control group. In the diaphyseal tibia, the periosteal circumference was significantly smaller in the CMA-refuted group compared with the control group, in the proximal tibia, the periosteal circumference was significantly smaller in both the CMA-confirmed group and the CMA-refuted group as compared with the control group (table 3). The Z-score for the periosteal circumference in the diaphyseal tibia was more frequently below −2.0 in the CMA-refuted group (39.5%) compared with the control group (14.6%) (p=0.037).

Group comparisons of raw bone characteristics values by ANCOVA were roughly similar (online supplemental tables 1 and 2). Associations between height and pQCT-derived vBMD parameters are presented in online supplemental table 3. The use of ICSs did not affect the results, data not shown.

### Body composition

The CMA-confirmed group had a significantly higher fat-free mass compared with the control group by bioelectrical impedance analysis. This difference was not significant in a stratified sex-specific analysis. Height SD scores or BMI-for-age did not differ between groups (table 1).

## Discussion

Our longitudinal study found reduced pQCT-measured vBMD in both the radius and the tibia and lower tibial BMC among adolescents with a history of AE and CMA (CMA-confirmed group) during early childhood compared with control groups. Body composition was similar across groups. These findings suggest a long-term effect of early childhood CMA and milk elimination diet on bone health in adolescents.

Our pQCT findings agree with a recent study on bone structure by high-resolution pQCT in participants with persistent IgE-mediated CMA, which found in the distal tibia, in postmenarchal females compared with controls, a reduced total and trabecular vBMD. No difference was observed in vBMD among premenarchal females. Noteworthy, approximately half of both participants and controls were vitamin D deficient (serum concentration <50 nmol/L),^15^ whereas most of our study participants were vitamin D sufficient^16^ (table 1). A distally reduced total vBMD could be explained by a reduced trabecular vBMD, as distal bone is primarily composed of trabecular bone. Trabecular vBMD was lowest in the CMA-confirmed group.

Our findings are important as reduced vBMD in childhood is associated with an increased fracture risk. In male adolescents (n=991, mean age 18.9 years), a decreased trabecular and cortical vBMD in both radius and tibia have been associated with a history of X-ray confirmed fractures.^24^ Children (n=224, mean age girls 10.1 years, boys 11.6 years) with radial and/or ulnar fractures have shown a reduced vBMD at the distal radius.^25^ We observed no increase in fracture rate. However, the observed differences in vBMD were modest at most measurement sites, except for the distal radius, where a clinically significant proportion of participants in the CMA-confirmed group had a Z-score below −2.0. Fractures associated with low childhood milk intake may not yet have manifested in the age group we studied. Further follow-up is necessary to determine the long-term clinical implications of our findings. In addition, our study cohort was rather small, and significantly larger cohorts would be needed to detect such differences. A study on the association between milk consumption in childhood (age 5–12 years) and adolescence (age 13–17 years) found in women aged ≥50 years (n=1880) an increased risk for osteoporotic fractures among women with a low childhood milk intake, but no association with fracture incidence in women aged 20–49 years.^26^

While we used pQCT, other studies have evaluated BMD in children with CMA by DXA. Postpubertal adolescents with persistent IgE-mediated CMA (n=33, mean age 19.7 years) have shown, compared with controls, a reduced aBMD Z-score at the hip, the femoral neck and lumbar spine. However, a successfully completed oral milk immunotherapy (n=12, mean age 20.2 years) increased aBMD compared with participants with persistent allergy.^27^ Our study found that vBMD was reduced in the CMA-confirmed group although all but one had achieved milk tolerance. A study that compared children with persistent CMA (n=52) to children with other food allergies (n=29) found a lower spinal aBMD by DXA associated with CMA, but not other food allergies.^13^ Our study found that participants with a history of IgE-mediated CMA had lower dairy consumption and lower vBMD compared with those with a history of non-IgE-mediated allergy. Furthermore, since most participants in the CMA-confirmed group had had an IgE-mediated allergy, our findings support the idea that differences in dairy product consumption may explain the observed differences in BMD. A positive relationship between calcium intake and bone mass accrual has been established in prepubertal children, and calcium supplementation enhances the positive effect of PA at skeletal sites that are weight bearing in young children,^11^ highlighting the importance of dietary calcium acquisition before the onset of pubertal maturation. This could explain why a milk elimination diet in early childhood may affect bone structure beyond the duration of the diet, as our current study shows.

We found a higher frequency of any purchase of ICS in the CMA-confirmed group, which is in line with our previous finding of a higher reported lifetime prevalence of wheezing in the CMA-confirmed group.^28^ Among children with CMA, low weight Z-score and diagnosis of asthma have been identified as independent risk factors for decreased aBMD.^29^ However, it is mainly high daily doses of ICS that affect bone loss, and adequate intake of vitamin D and calcium seem to mitigate these effects.^30^ In our study, a history of any ICS purchase was not associated with adolescent bone structure. The current national guidelines for asthma recommend treatment at low doses of ICS.

The strength of our study is the rigorous diagnosis of CMA by the gold standard method, as other studies often define CMA based on parental reports, IgE-mediated sensitisation or open oral food challenges. Another strength is the choice of bone structure analysis method, as it has been suggested that vBMD by pQCT, as opposed to aBMD by DXA, may provide more information about bone strength and fracture risk.^31^ Our study also has some limitations. Due to the cross-sectional design, we establish associations between CMA and reduced vBMD, but causality cannot be inferred. Another limitation is the lack of prospective data regarding the use of calcium substitutes during the span of the milk elimination diet, as well as on sunlight exposure, which may result in residual confounding that the adjusted model could not account for. The analysis on endocortical circumference Z-scores in the diaphyseal tibia should be interpreted with caution, as the median Z-score was low across groups. The calculation for Z-scores, which was inferred from a Belgian population, may be unsuitable for our Finnish population.

## Conclusion

In this cohort study, we found reduced vBMD in adolescents diagnosed with CMA during infancy. Most of the participants had an IgE-mediated allergy and these participants demonstrated lower total dairy consumption in adolescence. Our findings further support the interpretation that variations in long-term dietary habits, influenced by early-life CMA type and subsequent tolerance development, may have lasting effects on bone health trajectories. Reduced vBMD may influence fracture rate in older age, when the risk for osteoporotic fractures is at its highest. Our finding highlights the importance of starting a milk-elimination diet in early childhood only when CMA is challenge confirmed with clinically relevant symptoms. It is also fundamentally important to regularly reassess milk tolerance and actively introduce milk when tolerance has been achieved. For children with mild symptoms, desensitisation using the food ladder principle should be endorsed.^32^

## Supplementary material

10.1136/bmjpo-2025-004087 online supplemental table 1

## Data Availability

Data are available upon reasonable request.
