# Supplementary material for: Bone structure and body composition in adolescents with cow’s milk allergy in infancy: a clinical cohort study
Source: BMJ Paediatr Open. 2026 Jan 21;10(1):e004087. doi: 10.1136/bmjpo-2025-004087 (PMC12829375; doi:10.1136/bmjpo-2025-004087)
Supplement: online supplemental table 1 [file bmjpo-10-1-s001.docx]

| **Supplement table 1.** Bone structure as adjusted raw values in the radius among adolescents with atopic eczema and confirmed cow’s milk allergy during infancy, adolescents with atopic eczema and refuted cow’s milk allergy during infancy, and controls. | | | | | | | |
| --- | --- | --- | --- | --- | --- | --- | --- |
|  | CMA-confirmed  (n=38) |  | CMA-refuted  (n= 32) |  | Controls  (n= 46) |  | *P* |
|  | Mean | 95% CI | Mean | 95% CI | Mean | 95% CI |  |
| Distal radius (4% site) |  |  |  |  |  |  |  |
| Total BMC, mg/mm | 119 | 114-125 | 127 | 121-132 | 127 | 121-134 | 0.12 |
| Total CSA, mm^2^ | 405 | 388–423 | 395 | 377–413 | 410 | 390–430 | 0.51 |
| Total vBMD, mg/cm^3^ | 294 | 282–306 | 319 | 307–331 | 310 | 296–323 | **0.02^a^** |
| Trabecular vBMD, mg/cm^3^ | 194 | 182–205 | 211 | 199–223 | 204 | 191–218 | 0.12 |
| Proximal radius (66% site) |  |  |  |  |  |  |  |
| Total BMC, mg/mm | 106 | 102–109 | 103 | 99–107 | 109 | 105–114 | 0.11 |
| Total CSA, mm^2^ | 136 | 130–143 | 130 | 123–136 | 141 | 133–148 | 0.09 |
| Total vBMD, mg/cm^3^ | 786 | 761–811 | 801 | 775–827 | 785 | 756–814 | 0.63 |
| Cortical CSA, mm^2^ | 82.5 | 79.4–85.6 | 79.9 | 76.7–83.1 | 85.0 | 81.4–88.6 | 0.11 |
| Cortical vBMD, mg/cm^3^ | 1132 | 1124–1141 | 1138 | 1130–1147 | 1132 | 1122–1141 | 0.50 |
| Polar SSI, mm^3^ | 309 | 288–330 | 289 | 268–311 | 328 | 304–352 | 0.06 |
| CMA, cow’s milk allergy  ANCOVA adjusted for sex, supervised exercise during the past 5 years, age-adjusted body mass index, mean daily intake of dairy products, and mean daily intake of vitamin D from food and supplements.  ^a^ Groupwise comparison CMA-confirmed vs. CMA-refuted *P* < 0.05 | | | | | | | |

| **Supplement table 2.** Bone structure as adjusted raw values in the tibia among adolescents with atopic eczema and confirmed cow’s milk allergy during infancy, adolescents with atopic eczema and refuted cow’s milk allergy during infancy, and controls. | | | | | | | |
| --- | --- | --- | --- | --- | --- | --- | --- |
|  | CMA-confirmed  (n=38) |  | CMA-refuted  (n= 32) |  | Controls  (n= 45) |  | *P* |
|  | Mean | 95% CI | Mean | 95% CI | Mean | 95% CI |  |
| Distal tibia (4% site) |  |  |  |  |  |  |  |
| Total BMC, mg/mm | 336 | 321-352 | 345 | 329-362 | 372 | 355-390 | **0.009^a^** |
| Total CSA, mm^2^ | 1118 | 1063–1172 | 1070 | 1021–1118 | 1157 | 1105–1209 | **0.04^b^** |
| Total vBMD, mg/cm^3^ | 300 | 288–312 | 321 | 311–332 | 321 | 309–332 | **0.04^c^** |
| Trabecular vBMD, mg/cm^3^ | 239 | 228–251 | 250 | 240–260 | 256 | 246–267 | 0.14 |
| Diaphyseal tibia (38% site) |  |  |  |  |  |  |  |
| Total BMC, mg/mm | 351 | 333–370 | 351 | 334–368 | 379 | 362–397 | **0.04^c^** |
| Total CSA, mm^2^ | 403 | 381–425 | 393 | 373–412 | 429 | 408–449 | **0.03^b^** |
| Cortical thickness, mm | 5,12 | 4,91–5,34 | 5,26 | 5,07–5,38 | 5,42 | 5,21–5,62 | 0.19 |
| Cortical vBMD, mg/cm^3^ | 1149 | 1141–1158 | 1161 | 1153–1169 | 1156 | 1147–1164 | 0.17 |
| Endosteal circumference, mm | 38.6 | 36.8–40.4 | 36.9 | 35.3–38.5 | 39.1 | 37.4–40.8 | 0.12 |
| Periosteal circumference, mm | 70.8 | 68.9–72.7 | 69.9 | 68.2–71.6 | 73.2 | 71.3–75.0 | **0.03^b^** |
| Proximal tibia (66% site) |  |  |  |  |  |  |  |
| Total BMC, mg/mm | 380 | 366–395 | 387 | 371–402 | 421 | 405–438 | **0.001^a,b^** |
| Total CSA, mm^2^ | 601 | 575–628 | 583 | 554–612 | 656 | 625–686 | **0.002^a,b^** |
| Total vBMD, mg/cm^3^ | 642 | 621–663 | 670 | 647–692 | 647 | 623–671 | 0.19 |
| Cortical CSA, mm^2^ | 292 | 280–303 | 296 | 284–309 | 326 | 313–339 | **<0.001^a,b^** |
| Cortical vBMD, mg/cm^3^ | 1109 | 1102–1116 | 1121 | 1114–1129 | 1113 | 1105–1121 | 0.07 |
| Cortical thickness, mm | 3.94 | 3.79–4.09 | 4.10 | 3.94–4.25 | 4.21 | 4.04–4.37 | 0.06 |
| Endosteal circumference, mm | 61.6 | 59.4–63.8 | 59.4 | 57.1–61.8 | 64.0 | 61.5–66.6 | **0.04^b^** |
| Periosteal circumference, mm | 86.4 | 84.4–88.3 | 85.2 | 83.1–87.2 | 90.4 | 88.2–92.7 | **0.002^a,b^** |
| Polar SSI, mm^3^ | 2372 | 2225–2519 | 2363 | 2205–2520 | 2723 | 2555–2891 | **0.003^a,b^** |
| CMA, cow’s milk allergy  ANCOVA adjusted for sex, supervised exercise during the past 5 years, age-adjusted body mass index, mean daily intake of dairy products, and mean daily intake of vitamin D from food and supplements.  ^a^ Groupwise comparison CMA-confirmed vs. control, *P* < 0.05  ^b^ Groupwise comparison CMA-refuted vs. control, *P* < 0.05  ^c^ Groupwise comparisons non significant | | | | | | | |

| **Supplement table 3.** Correlations of various volumetric bone mineral density measurements and height. | | |
| --- | --- | --- |
|  | Correlation | *P* |
| Distal radius (4% site) |  |  |
| Total vBMD, Z-score | 0.07 | 0.42 |
| Trabecular vBMD, Z-score | 0.08 | 0.36 |
| Proximal radius (66% site) |  |  |
| Total vBMD, Z-score | -0.23 | **0.009** |
| Cortical vBMD, Z-score | -0.13 | 0.15 |
| Distal tibia (4% site) |  |  |
| Total vBMD, Z-score | -0.09 | 0.33 |
| Trabecular vBMD, Z-score | 0.01 | 0.90 |
| Diaphyseal tibia (38%) |  |  |
| Cortical vBMD, Z-score | -0.17 | 0.05 |
| Proximal tibia (66% site) |  |  |
| Total vBMD, mg/cm^3^ | -0.31 | **<0.001** |
| Cortical vBMD, mg/cm^3^ | -0.53 | **<0.001** |
