# Supplementary material for: Bone structure and body composition in adolescents with cow’s milk allergy in infancy: a clinical cohort study
Source: BMJ Paediatr Open. 2026 Jan 21;10(1):e004087. doi: 10.1136/bmjpo-2025-004087 (PMC12829375; doi:10.1136/bmjpo-2025-004087)
Supplement: Supplementary file 2 [file bmjpo-2025-004087.reviewer_comments.pdf]

PEER REVIEW HISTORY

BMJ Paediatrics Open publishes all reviews undertaken for accepted manuscripts. Reviewers are asked to complete a checklist review form and are provided with free text boxes to elaborate on their assessment. These free text comments are reproduced below.

ARTICLE DETAILS

**TITLE (PROVISIONAL)**	Bone structure and body composition in adolescents with cow's milk allergy in infancy: a clinical cohort study
**AUTHORS**	Piippo, Sonja; Varimo, Tero; Hauta-alus, Helena; Viljanen, Mirva; Savilahti, Erkki; Mäkitie, Outi; Kuitunen, Mikael

VERSION 1 - REVIEW

**REVIEWER NAME**	Lena Wallensteen
**REVIEWER AFFILIATION**	Karolinska Institutet, Womens and Childrens health
**REVIEWER CONFLICT OF INTEREST**	N/A
**AI DISCLOSURE**	No
**DATE REVIEW RETURNED**	17-Oct-2025

**GENERAL COMMENTS**	The study has limitations due to its design. A retrospective study based on individuals recruited for a study with other aims and objectives is challenging. There are several important data missing as previous reviewers already mentioned. This is something that I think is important to discuss further in the manuscript.<br><br>1) How long was the period of milk exclusion? In previous answer, to reviewer 1, you stated that the median duration was 2y and the range was 0-11y. However, this question was only answered by 60% of the individuals in that group. This is an important fact, and I think it should be addressed in the manuscript not only in the answer to the reviewer. (Maybe in the table with characteristics of participants?)<br>2) The controls were not matched for sex which is a problem since females usually have an earlier puberty, a higher percentage of body fat and may have different kind/amount of physical activity than boys in this age group. All these aspects effect the BMD. Why did you not recruit more boys?<br><br>3) Consider including the tanner stage as part of the characteristics in table 1. However, the age at onset of puberty is maybe more accurate to evaluate since a late onset of puberty may have a big impact on BMD. This is difficult to investigate retrospectively, especially in boys, but did you consider this in the interpretation of the results?<br><br>4) In the abstract, you may consider changing the statement in conclusion to a more descriptive approach: The conclusion that "early childhood CMA "IS" associated with lower adolescent radial and tibial vBMD" should, in my opinion, be changed to "MAY BE" and that further studies are needed.

**REVIEWER NAME**	Junichiro Tezuka
**REVIEWER AFFILIATION**	Fukuoka Children's Hospital, Allergy and Pulmonology
**REVIEWER CONFLICT OF INTEREST**	N/A
**AI DISCLOSURE**	No
**DATE REVIEW RETURNED**	03-Nov-2025

**GENERAL COMMENTS**	General Comments<br>This manuscript presents a unique and clinically valuable long-term follow-up study investigating bone structure and body composition in adolescents with confirmed cow's milk allergy (CMA) during infancy. The study benefits from a well-characterized cohort in which CMA diagnosis was rigorously established by double-blind placebo-controlled food challenge (DBPCFC), and from the use of pQCT-derived volumetric bone density (vBMD) rather than areal BMD.<br>The authors have addressed most of the previous reviewers' concerns comprehensively and transparently. The revised version now provides a clearer description of the statistical approach, explicitly defines the exposure–outcome relationship analyzed with ANCOVA, and includes sex as a covariate. The abstract and results are simplified and aligned with the adjusted model, improving readability and scientific precision. Overall, the revisions substantially strengthen the manuscript's methodological clarity and clinical interpretability.<br>The findings—that early-life CMA, even when later resolved, is associated with lower vBMD at the distal radius and tibia—are biologically plausible and clinically relevant. Despite some inherent limitations, this paper provides an important contribution to the literature on long-term nutritional and skeletal outcomes following early elimination diets.<br><br>Major Comments<br>1. Residual uncertainty in causal interpretation<br>While the authors correctly avoid causal overstatements, it may be helpful to emphasize in the Discussion that, despite adjustment for major confounders (sex, BMI, physical activity, and vitamin D intake), residual confounding by unmeasured early-life factors (e.g., total calcium intake, duration of elimination, sunlight exposure) cannot be excluded. This will reinforce the appropriately cautious tone of the conclusions.<br>2. Interpretation of clinical significance<br>The authors mention that fracture rates were not increased in this adolescent sample. It would strengthen the Discussion to explicitly note that although the vBMD Z-scores are below average, the magnitude of difference is modest and its long-term clinical impact remains to be determined, especially as the cohort ages.<br>3. Clarify extent of "current milk consumption"<br>The text states that only one subject continued milk avoidance at follow-up. It might be useful to specify whether the current milk/dairy intake among CMA-confirmed participants reached a comparable quantitative level (e.g., servings/day) to controls, as this relates directly to the interpretation of persistent vBMD differences despite similar current intake.

VERSION 1 – AUTHOR RESPONSE

Reviewer: 1

Comments to the Author

1. What is the implication or possible reason that BMD is reduced in one bone but not another?

RESPONSE: Thank you for this question. The BMD was reduced in the radius and the tibia, both bones measured. Observed differences between measurement sites may be related to the fact that the tibia is a weight-bearing bone. A milk intake that provides adequate dietary calcium may amplify the effect of physical activity on BMD and bone strength. Thus, a milk elimination diet might reduce the beneficial effects of exercise on BMD, which is more likely to be seen in the weight-bearing bones.

Due to structural differences between bone compartments, the effects of milk consumption may vary across them. Distal bone sites are primarily composed of trabecular bone. The calcium content in trabecular bone is lower than in cortical bone, while 80% of bone mass exists in the cortical compartment. However, trabecular bone has a surface-to-volume ratio nearly 10 times greater than that of cortical bone, making it more metabolically active and potentially more responsive to dietary influences such as calcium intake from milk. It has been proposed that decreased bone density due to inadequate calcium intake might occur sooner in trabecular than cortical bone. This could explain the reduced vBMD at the distal measurement sites in both bones.

Please see Taylor C. Wallace, Regan L. Bailey, Joan Lappe, Kimberly O. O'Brien, Ding Ding Wang, Shivani Sahni & Connie M. Weaver (2021) Dairy intake and bone health across the lifespan: a systematic review and expert narrative, Critical Reviews in Food Science and Nutrition, 61:21, 3661-3707, DOI: 10.1080/10408398.2020.1810624

Susan M. Ott; Cortical or Trabecular Bone: What's the Difference?. Am J Nephrol 11 July 2018; 47 (6): 373–375. https://doi.org/10.1159/000489672

2. How long since the children outgrew their CMA – did that have an effect on BMD e.g. some children might have continued milk avoidance, and some might have introduced a lot of milk

RESPONSE: Thank you for this important notion. The potential lifetime differences in milk consumption between those with CMA and those who did not have CMA may be the cause of the observed differences in BMD between groups. In the CMA-confirmed group with a measured BMD, 26 participants (60%) have disclosed their age at which milk was reintroduced in the diet. The median duration of the elimination diet was 2 years in this group, the range being 0-11 years. To explore the effect of the elimination diet duration on vBMD, correlations were calculated. In the radius, elimination diet duration showed only weak negative correlations with vBMD Z-scores, none of which were statistically significant (all $P > 0.05$). In the tibia, elimination diet duration showed modest negative associations with total and trabecular vBMD at the distal measurement site, as well as with cortical vBMD at the diaphyseal measurement site, though these were not statistically significant. These findings were not included in the manuscript to maintain clarity.

3. You collected current diet intake using FFQs – did you take current intake e.g. calcium and Vitamin D into account? I noticed you took currently milk intake into account.

RESPONSE: Thank you for this observation. We have previously reported that there were no differences in the intake of Vitamin D from food nor supplements. Piippo, S., Hauta-alus, H., Viljanen, M. et al. Dairy consumption and vitamin D concentration in adolescents with challenge-confirmed cow's milk allergy during infancy. Eur J Clin Nutr 78, 897–904 (2024). https://doi.org/10.1038/s41430-024-01477-x

As the majority of calcium intake is from dairy products, we interpreted daily dairy intake as a proxy for dietary calcium intake. Our previous model only adjusted for age-adjusted BMI and supervised physical activity during the past 5 years. As per your suggestion we now did a new analysis of covariance that adjusted for sex, age-adjusted BMI, supervised physical activity during the past 5 years, daily dairy product consumption, and daily intake of vitamin D combined from food and supplements. We replaced the results for the adjusted model in tables 2 and 3 with the updated results from this new analysis, as well as updated methods on page 6 "We compared pQCT-derived bone parameters from the three groups by ANCOVA to adjust for potential confounders. The final model included the covariates sex, BMI-for-age, supervised PA during the past 5 years, daily dairy product consumption, and daily intake of vitamin D from food and supplements. Pairwise comparisons were Bonferroni-corrected.", and results on page 8-10. "In the adjusted model in the CMA-confirmed group, total vBMD was significantly lower at the distal radius compared to the CMA-refuted group, and in the distal tibia compared to the control group", "Cortical vBMD in the diaphyseal tibia was lower in the CMA-confirmed group compared to the CMA-refuted group.", "In the adjusted model, the total cross-sectional area was smaller in the CMA-refuted group compared to controls in the proximal radius as well as all three tibial measurement sites. The CMA-confirmed group exhibited a smaller cross-sectional area compared to controls in the proximal tibia."

4. Do you have any data on which milk replacement/hypoallergenic formula the milk allergic children used or of they were breastfed? You may have to take this into account in your models.

RESPONSE: Thank you for this question. Unfortunately, we do not have data on the type of formula that was used in the treatment of the children with CMA, nor on the duration of their breastfeeding. However, the majority have after weaning been treated with an extensively hydrolyzed formula as amino-acid formulas were not readily available at the time-period of their early childhood.

5. What % children had IgE or non-IgE mediated CMA and did that affect BMD?

RESPONSE: Thank you for this valuable question. In the CMA-confirmed group among those with a measured BMD, 24 participants (56%) originally had an IgE-mediated CMA, and 19 participants (44%) had a non-IgE-mediated CMA. A comparison of these two subgroups with a Mann Whitney-U analysis showed a significant difference in the distal radius Z-score of trabecular vBMD, in the distal tibia Z-score for trabecular vBMD as well as total vBMD. We also conducted a comparison of differences in current daily dairy product consumption between those with an IgE-mediated CMA and those with a non-IgE-mediated CMA, which showed a significant difference. These findings agree with the idea that a difference in dairy product consumption may be the explanation for the observed differences in BMD. This further supports the interpretation that variations in long-term dietary habits, shaped by early-life CMA type and subsequent tolerance development, may have lasting effects on bone health trajectories. These findings were added on page 8 "There was no difference in total dairy product consumption between the groups. 16 A sub-analysis within the CMA-confirmed group revealed that participants with a history of IgE-mediated CMA had a lower median total daily dairy product consumption compared to those with a history of non-IgE-mediated CMA (317 g/d vs. 661 g/d, respectively) (P=0.04). ", page 9 "A sub-analysis within the CMA-confirmed group found that participants with IgE-mediated CMA had a lower median Z-score compared to those with non-IgE-

mediated CMA for distal radius, trabecular vBMD (-0.59 vs. 0.32, P=0.03), the distal tibia total vBMD (-0.08 vs. -0.01, P=0.01) and trabecular vBMD (-0.18 vs. 0.46, P=0.01). and subsequently added in the discussion on page 12 “Our study found that participants with a history of IgE-mediated CMA had lower dairy consumption and lower vBMD compared to those with a history of non-IgE-mediated allergy. Furthermore, since the majority of participants in the CMA-confirmed group had had an IgE-mediated allergy, our findings support the idea that differences in dairy product consumption may explain the observed differences in BMD. “ and conclusions p. 14 “Most of these participants had an IgE-mediated allergy and demonstrated lower total dairy consumption in adolescence. These findings further support the interpretation that variations in long-term dietary habits, influenced by early-life CMA type and subsequent tolerance development, may have lasting effects on bone health trajectories.”.

6. Was there a difference in length between the 3 groups?

RESPONSE: Thank you for this question. As reported in table 1, there was no difference between groups in height measured in centimeters nor when compared as standard deviation scores.

7. The CMA group had a higher use of corticosteroids, did that have any effect on your outcomes?

RESPONSE: We are grateful for this question. We previously did an analysis of covariance for the pQCT parameters which adjusted for the use of ICS along with age-adjusted BMI and daily mean supervised PA during the past 5 years. The inclusion of this covariate did not change the results, and the use of ICS did not reach statistical significance in the model. We omitted this covariate from the final model as we did not want to reduce the statistical power of the analysis. We have summarized this finding on page 10. ”The use of inhaled corticosteroids did not affect the results, data not shown.”

8. Did length or BMI show any association with BMD?

RESPONSE: Thank you for this question. We examined associations of height and age-adjusted BMI with pQCT-derived BMD parameters. Correlations with height were generally weak, although modest negative associations were seen with vBMD at the proximal tibia ($P < 0.001$). Age-adjusted BMI showed modest positive associations with total and trabecular vBMD at the distal radius and tibia ($P < 0.01$), whereas correlations at other sites were weak and not statistically significant. As these associations suggest that body size only had a limited influence on BMD in our cohort, findings were added in the supplement table 3, and this was added in the manuscript on pages 9-10 ”Associations between height and pQCT-derived vBMD parameters are presented in Supplemental Table 3.”

9. Why did you not take current calcium and vitamin D status into account in your models?

RESPONSE: Thank you for this important question. We did not originally include these parameters as covariates in the analysis of covariance as current daily dairy consumption (a proxy for dietary calcium intake) and vitamin D status did not differ between groups, and we did not want to compromise the statistical power of the test. We have now updated the model, please see question 3. Including both the 25(OH)D concentration and vitamin D intake from food and supplements would lead to issues with multicollinearity, as they are correlated (Spearman’s correlation 0.38 ($P<0.001$))

10. You may also want to assess overall diet (kcal and macronutrients) and its association with BMD.

RESPONSE: Thank you for your thoughtful suggestion regarding the assessment of overall diet (kcal and macronutrients) in relation to BMD. While we agree that dietary intake can be an important factor, incorporating a detailed nutritional analysis would require substantial additional data collection and processing, which is beyond the scope of the current study. We appreciate the insight and will consider this direction for future research.

Reviewer: 2

Comments to the Author

This is a cross-sectional follow-up of young people who underwent double-blind placebo controlled oral food challenge by age 12 months. 104 of the original cohort consented to follow-up at 15-18 years: 43 has milk allergy at 12 months of age, 38 did not; a third group of 49 non-milk-allergic controls were also recruited. The authors report that those with milk allergy at age 12 months had lower adolescent radial and tibial vBMD.

These findings are difficult to interpret due to the following reasons;

- no data are presented in terms of how long participants were excluding milk from their diet... if the hypothesis is that CM-exclusion is associated with impaired BMD, then this would be a critical supportive finding - something which should be possible in the context of the current study without major impact of recall bias.

RESPONSE: Thank you for raising this important point. As this issue was also addressed in our response to Reviewer 1 (Comment 2), to avoid repetition we refer you to our response there, where we provide a detailed rationale.

- there is no assessment of the milk-alternative used in the CM-allergic participants, and whether additional Ca/Vit D supplementation was used (which is near-mandatory now). This might be possible, although recall bias may be a concern.

RESPONSE: We appreciate your comment. The issue regarding milk-alternatives used in the treatment of participants with CMA was also raised by Reviewer 1 (comment 4). We kindly refer you to that response for a detailed explanation. Data on the use of Ca/Vit D supplementation during the time of the milk elimination diet was not collected as the likelihood of recall bias was deemed very high.

- a key factor impacting on bone density is exercise: the authors did try to incorporate this as a possible confounder, but could only do so through estimates of "any physical activity" over the past 1/5 years.

RESPONSE: We are thankful for this insightful comment and fully agree that exercise is an important factor to consider when analyzing BMD. This is why participants in the questionnaire reported all different sport disciplines under supervised training that they had participated in during the past 12

months and during the past 5 years, as well as how many months during the year and how many times during a week this sport was practiced, and the length of a single session. They similarly reported unsupervised exercise that included walking, running, swimming, bicycling, and trekking. The questionnaire also assessed time spent walking/bicycling to and from school as well as whether the participant had participated in school PA. From this quite detailed data we calculated separately supervised and unsupervised PA for both the past 12 months and past 5 years. The variable supervised PA during the past 5 years was included in the analysis of covariance, as it showed the strongest correlations with vBMD parameters. Due to issues with multicollinearity only this one variable that represented the exercise history of the participant was included as a covariate in the final model.

These major confounders lead me to believe that at best, these data are hypothesis-generating and cannot be used to infer any conclusions. Prospective follow-up of a case-control cohort, with BMD assessed at regular intervals, is needed.

RESPONSE: Thank you for your comment. Our study was exploratory in nature, aiming to generate new insights into pQCT-derived vBMD in adolescents with a history of CMA, an area where existing research is scarce. We agree that future studies should validate these findings through prospective designs that include detailed data on dairy consumption, calcium and vitamin D supplementation, and regular pQCT assessments of vBMD.

Reviewer: 3

Comments to the Author

1. Abstract wording of results are difficult to follow in their current format. Perhaps remove the "comparing the three groups" wording , and simply state "The CMA-confirmed group had lower vBMD at the distal radius compared with the CMA-refuted group (Z-scores -1.49 vs -0.78)." The second sentence is easier to follow if written more simply as "The CMA-confirmed group had lower median total vBMD at the distal tibia compared with controls (Z-scores -0.05 vs +0.01). Lower median trabecular vBMD was observed after adjusting for past 5 years' mean daily supervised physical activity and age-adjusted body mass index (Z-scores +0.20 vs +0.51).

RESPONSE: Thank you for these valuable suggestions. The abstract has been revised accordingly. It has also been revised to report the results of the updated adjusted model, that was done based on reviewer 1 comments. Please see page 1 for the updated abstract.

2. Please clarify in the abstract text if the first two results mentioned are unadjusted or adjusted, if adjusted then what for?

RESPONSE: Thank you for highlighting this matter. The first two results are true for both the unadjusted and adjusted model. To clarify the main results as per the suggestion in your comment 7 the abstract has now been updated to include the results from the adjusted model only.

*Overall statistical approach and reporting

The overall approach to analyses is relatively descriptive and not clearly reported - which hampers the interpretation of the findings.

In terms of reporting I have the following comments:

3. It is good practice to clearly state the direct application and purpose of each statistical method in the context of the study this helps orientate the reader around the methods and whether they are appropriate , as well as improves reproducibility of the science. For example "we assessed categorical variables by Chi-Squared and Fishers exact test" requires details what was assessed [e.g. differences in "what" (BMD?), between which groups (people with and without cow milk allergies and controls?) for which type of categorical variables (e.g. participant demographics and other study measurements?). The same applies to "results presented as medians and IQRs" (which results specifically?) and "ANCOVA was performed to adjust for potential confounders" (ANCOVA was performed to assess the association of what on what?).

RESPONSE: Thank you for your comment. We have revised the reporting of statistical analyses in the methods section based on your suggestions on page 6 “Differences in categorical background characteristics between the CMA-confirmed group, the CMA-refuted group and controls were analysed by $\chi 2$, except for family history of osteoporosis which was analysed by Fisher’s exact test” and “ANCOVA was performed to assess the association between a history of CMA during infancy on vBMD in adolescence, controlling for covariates.”

In terms of analyses approach and interpretation I have the following comments:

4. The authors report that a final model was identified based on clinical relevance among covariates with significant correlations. Considering the authors are not aiming to develop a prediction model according to my understanding of the work (e.g. prediction modelling would imply that prediction accuracy is the main priority and causal relations between variables are not) - all clinically relevant variables which are potential confounders of the exposure/outcome relationship regardless of their univariate correlations should be considered for inclusion in the model. Without this there is potential for a high degree of bias given that potential confounders are excluded.

RESPONSE: Thank you for this suggestion. The original model included as few covariates as possible as to not lose statistical power. As suggested, we have taken a more clinical approach to the covariates included. The details of the updated model are provided in our response to Reviewer 1 (Comment 3), to avoid repetition we refer you to our response there, where we provide a detailed rationale.

5. There is a large mismatch in terms of the gender distribution between the study arms and the age-matched controls which is problematic for interpretation of findings. At the very least it strikes me as critical to include sex in all of the core analyses - which does not currently appear to be the case and is likely to cause significant potential for bias.

RESPONSE: Thank you for this important comment. This is the reason why we primarily explored the Z-scores of the pQCT-derived bone parameters that were adjusted for sex. We have now also included sex as a covariate in the adjusted model. The details of the updated model are outlined in our response to Reviewer 1 (Comment 3), to avoid repetition we refer you to our response there, where we provide a detailed rationale.

6. I couldn't find clear rationale for analysing three groups of bones in the methods which needs to be added. Analyses has been conducted, as far as I could see, stratified by these three types. A hierarchical model that takes into account dependencies and relationships between data from the same individuals on different bones would be required to provide more accurate estimates and improve statistical power - if I have understood the study design correctly.

RESPONSE: Thank you for this suggestion. We have not analyzed three groups of bones but rather two bones. Data from different measurement points in the same individual were not compared to one another, thus we did not deem it necessary to consider within-participant relationships.

7. The emphasis on unadjusted findings over adjusted findings is of concern and is likely to lead to misinterpretation of the study. As per reporting standards - it is important to show unadjusted estimates alongside adjusted ones - however - it is not appropriate to then present the unadjusted findings as the key study outputs. The adjusted findings (if appropriately adjusted) are the ones that should be "final" and form the main part of the results and discussion.

RESPONSE: Thank you for your comment. Our rationale was that as our data on bone parameters was non-normally distributed, Kruskal-Wallis is the most appropriate test to find differences between study groups. As there are many potential covariates that may influence the results, we wanted to additionally compare bone parameters with ANCOVA, even as there were minor violations of the assumptions. Based on your suggestion, we have revised the results and discussion with the adjusted results as our primary and final result on pages 8-9 ”In the adjusted model in the CMA-confirmed group, total vBMD was significantly lower at the distal radius compared to the CMA-refuted group, and in the distal tibia compared to the control group (Figure 2). In the distal radius, but not at other measurement sites, the proportion of vBMD Z-scores below -2.0 was higher in the CMA-confirmed group (37.2%) compared the CMA-refuted (5.3%) and control groups (14.6%) (P=0.001). Trabecular vBMD in the distal tibia was lower in the CMA-confirmed group compared to the control group. (Figure 2) Cortical vBMD in the diaphyseal tibia was lower in the CMA-confirmed group compared to the CMA-refuted group. (Table 3)

In the adjusted model BMC was significantly lower in the CMA-confirmed group compared to controls at the distal radius, and in both the CMA-confirmed group and the CMA-refuted group compared to the control group at all three tibial measurement sites (Table 3). At the diaphyseal tibial site BMC Z-score was more frequently below -2.0 both in the CMA-confirmed group (11.6%) and the CMA-refuted group (13.2%), compared to the control group (0%) (P=0.038). A sub-analysis within the CMA-confirmed group found that participants with IgE-mediated CMA had a lower median Z-score compared to those with non-IgE-mediated CMA for distal radial trabecular vBMD (-0.59 vs. 0.32, P=0.03), the distal tibial total vBMD (-0.08 vs. -0.01, P=0.01) and trabecular vBMD (-0.18 vs. 0.46, P=0.01).

In the adjusted model the total cross-sectional area was smaller in the CMA-refuted group compared to controls in the proximal radius as well as all three tibial measurement sites. The CMA-confirmed group exhibited a smaller cross-sectional area compared to controls in the proximal tibia. (Table 3).

In the adjusted model, the endosteal circumference at the diaphyseal and proximal tibial measurement site was significantly smaller in the CMA-refuted group compared to the control group.”

8. The language use for interpretation of findings and differences in significance between various parts of the analyses is problematic. For example the authors state "this difference lost significance in

a stratified sex-specific analysis." Significance is not really gained or lost - a particular result is either significant or not. If an unadjusted analyses resulted in a significant finding, and the subsequent adjusted analyses did not show statistical significance then a better way to present this would be to suggest that after taking account of confounders - there was no significant difference between X & Y.

RESPONSE: Thank you for this insightful suggestion. The results section on page 8-10 has been revised accordingly.

9. Table 1 includes a number of cells which have data pertaining to small numbers (fewer than 5 individuals in some groups). Small number suppression is usually required or mandated in ethics applications or by data providers as small counts are considered potentially identifiable data - please check you are able to share such small counts openly or apply small number suppression throughout the presented work.

RESPONSE: Thank you for this question. We have verified this is not a concern.

10. Table 1 summarises some missing data - but there is no mention of this in the methods and how any bias from missing data may have been (or not) addressed in analyses. Please include.

RESPONSE: Thank you for raising this important point. Missing data was handled with pairwise deletion. This information has been added in the methods section on page 6. “Missing data were handled using pairwise deletion, allowing all available data to be used in each analysis.”

11. Throughout your tables and figures please be clear as to what is being presented. In table 1 please specify the values reported e.g. Median and IQR for age, N (%) for sex etc directly in the table. I think the "unless otherwise stated" part is not clear enough for the reader and think this is better presented in a column or next to the name of each variable.

RESPONSE: Thank you for this valuable suggestion. Table 1 has been updated according to your suggestion.

12. Add a sub-header to tables 2 & 3 which states that the values are Z-score medians and Q1,Q3 rather than a footnote.

RESPONSE: Thank you for this suggestion. Sub-headers have been added to tables 2 and 3. However, in table 3 the sub-header becomes quite long, as the proximal measurement site is not presented as median but as mean (95% confidence interval).

13. Figure 1 - include %s next to numbers in the flow diagram to better understand the proportion included/excluded at each stage

RESPONSE: Thank you for this comment. Figure 1 has been updated accordingly, with the proportion of participants included from the previous stage to the next.

14. Figure 2 - panel B (does not indicate which comparison was made and requires a p-value even if not significant). Please consider adding actual values to these plots to have a better understanding of the data being compared (e.g. in a jitter plot). Please clarify if these are representing findings from adjusted or unadjusted analyses.

RESPONSE: Thank you for this observation. We have now added P values from the adjusted model for all the pairwise comparisons made, both significant and non-significant. We also greatly appreciate jitter plots; thus, it was our primary intention to present the plot in this way. Unfortunately, the software available at our unit did not properly interpret the negative Z-scores and as such we were not able to construct a jitter plot. We have updated the figure legend to better describe the results that are presented. Please see figure 2 and figure legend 2 for the revised versions.
